# Chemoradiation after chemotherapy for metastatic cervical cancer can provide chemo-free time

**DOI:** 10.3389/fonc.2026.1826017

**Published:** 2026-06-02

**Authors:** Spoorthi Kamepalli, Ashley Chavana, Michelle Ludwig, Madeline Flanagan, Shelly Sharma, Tracilyn Hall, Anthony Costales, Jan Sunde, Claire Hoppenot

**Affiliations:** 1 Baylor College of Medicine, Houston, TX, United States; 2 Department of Radiation Oncology, Dan L Duncan Comprehensive Cancer Center, Baylor College of Medicine, Houston, TX, United States; 3 Department of Gynecologic Oncology, Dan L. Duncan Comprehensive Cancer Center, Baylor College of Medicine, Houston, TX, United States

**Keywords:** cervical cancer, chemoradiation, metastatic cervical cancer, palliative radiation, quality of life

## Abstract

**Introduction:**

Stage IVB cervical cancer is treated with a life-prolonging and palliative intent with systemic chemotherapy and immunotherapy. The purpose of this study was to evaluate the number of these patients who had an adequate response to chemotherapy to be eligible for consolidative radiosensitizing chemotherapy and radiation (chemoradiation) and subsequent outcomes.

**Materials and methods:**

This is a retrospective review of metastatic cervical cancer patients treated with chemotherapy and evaluated for treatment with consolidative pelvic chemoradiation at a single safety-net hospital system from 2016 to 2024. Demographic data and clinical information were collected from electronic medical records. Stata was used for descriptive statistical analysis.

**Results:**

38 patients met inclusion criteria. Most patients (84%) had squamous cell histology. All patients received platinum-based chemotherapy as first line treatment, some with triplet therapy with immunotherapy (26%) or bevacizumab (42%). Twenty (53%) patients received consolidative chemoradiation. These patients had a median treatment-free interval of 13.7 months (Interquartile range (IQR) 5.3-36.5) after finishing radiation, and 7 (35%) had no evidence of disease at last follow up. Overall survival was 26.7 months (IQR 16.8-55.9). Eighteen patients (47%) progressed on first line chemotherapy and were not eligible for chemoradiation. These patients had a median overall survival of 14 months (IQR 9.1-17.6). Factors at initial presentation associated with progression of disease on first line chemotherapy and ineligibility for chemoradiation prescription for pain medications before treatment (Odds Ratio (OR) 0.17, p=0.059), and prescription for anticoagulation for venous thromboembolism prior to treatments (OR 0.02, p=0.046).

**Conclusion:**

Consolidative pelvic radiation may be considered as a treatment option for select patients with stage IVB cervical cancer. Further research is needed to identify optimal chemoradiation regimens for local disease control, improvements in quality of life and improved health outcomes in the management of metastatic cervical cancer, particularly given the increasing use of pembrolizumab as maintenance therapy.

## 1 Introduction

Cervical cancer is the fourth most common cause of cancer in women worldwide, causing 348,189 deaths world-wide in 2022 (1). It is the most diagnosed cancer in 23 countries and leading cause of death in 36 countries (1). Despite improvements in screening and treatments over time, cervical cancer mortality remains high, particularly for those with advanced disease (2). Overall survival (OS) at 2 years in a 2024 study was 40-50% (3).

Both radiation and chemotherapy have been key treatment modalities for cervical cancer, though most commonly used in early stage cancers as concurrent chemoradiation, induction chemotherapy with subsequent chemoradiation in the Interlace trial, or combination of immunotherapy with chemoradiation in Keynote A18 (4–6). However, The primary treatment modality for stage IVB cervical cancer is systemic chemotherapy alone (7). Over the years, new treatment options for recurrent/metastatic cervical cancer have emerged. Notably biomarker-based immunotherapy agents like pembrolizumab with or without bevacuzimab have been added to chemotherapy regimens (8). Despite these new therapeutic interventions, a meta-analysis of the GOG 240, KEYNOTE-826, and BEATcc studies found poorer PFS and OS for patients following treatment of stage IVB cervical cancer compared to persistent and recurrent disease (2). Although there is significant research on the earlier stages of cervical cancer, fewer studies have solely examined patients with advanced stage disease, highlighting the need for further research on this patient population (2, 9).

In recent years, several studies have found that local therapies may be associated with improved survival in patients with metastatic cancers (10, 11). For metastatic cervical cancer in particular, a study by Wang et al. found that newly diagnosed metastatic cervical cancer treated with consolidative pelvic radiation therapy and chemotherapy was associated with longer patient survival than treatment with chemotherapy alone (12). Likewise, a smaller study by Wu et al. demonstrated superior PFS in metastatic cervical cancer patients treated with radiosensitizing cisplatin with radiation (chemoRT) compared to those treated with chemotherapy alone (13).

Given the sparsity of literature on clinical outcomes in stage IVB cervical cancer specifically, further research is needed to determine the best treatment practices for this patient population. The primary objective of this retrospective review of patients with metastatic cervical cancer is to evaluate rates of candidacy and outcomes for concurrent chemotherapy and radiation in patients after initial treatment with systemic regimens.

## 2 Materials and methods

This is a retrospective chart review of 38 FIGO stage IVB cervical cancer patients who were treated with chemotherapy and evaluated for treatment with consolidative chemoRT at a single safety-net hospital system in Houston, Texas, from 2016 to 2024. This study was approved by the Institutional Review Board (IRB) at Baylor College of Medicine (H-54535). Adult patients were included if they had stage IVB cervical cancer at time of diagnosis and if they received at least one cycle of systemic chemotherapy with or without targeted treatments or immunotherapy as the initial treatment modality. Patients who were unable to receive at least one dose of systemic treatments were excluded. Demographic and clinical data including imaging, treatment details, and reasons for treatment changes were collected from electronic medical records.

Patients were evaluated per individual physician criteria regarding eligibility for chemoRT. Generally, patients with complete response of disease outside of the radiation field with adequate performance status were referred to chemoRT. Some patients with partial response of extra-pelvic metastases were evaluated for stereotactic radiation to residual metastatic disease in addition to consolidative pelvic radiation. Patients with progressive disease, particularly if outside of the planned radiation field, were transitioned to second-line systemic treatments and were not offered chemoRT.

The primary outcome of this study is the rate of response to chemotherapy (as measured by patient eligibility for consolidative chemoradiation) and time without treatments after chemoradiation. Secondary outcomes were OS and PFS. Continuous variables were reported as a median with range. A logistic regression model was used to perform univariate analysis of various factors affecting patient eligibility for chemoRT treatment. All analyses were performed using Stata, version 13.1 (Stata Corp, College Station, TX).

## 3 Results

See **Table 1** for demographic and histopathologist characteristics. A total of 38 patients met inclusion criteria; all had stage IVB disease, 31 (82%) were Hispanic, with a median age of 49 years. Most patients (84%) had squamous cell histology. All patients received platinum-based chemotherapy as first line treatment (**Table 2**), and a minority received triplet therapy with immunotherapy (26%) and/or bevacizumab (42%). Of the 10 patients who received immunotherapy, only 3 were then eligible for consolidative chemoradiation, and none received immunotherapy during chemoradiation or as maintenance. The median number of cycles prior to progression or consolidative chemoradiation was 6 cycles (IQR 4-7) of platinum-based chemotherapy. Eight patients (21%) required palliative radiation due to bleeding or pain, and none of these patients became eligible for consolidative chemoradiation.

**Table 1:** Demographics and histopathologic characteristics.

Demographic factors	Total (N = 38)	No ChemoRT (N = 18)	ChemoRT (N = 20)	
Age	49 (32-71)	48 (32-71)	51 (34-67)	p=NS
BMI at diagnosis	28 (17-42)	27 (17-42)	28 (19-38	p=NS
Race/Ethnicity				
Hispanic	31 (82%)	15 (83%)	16 (80%)	
Black	6 (16%)	3 (17%)	3 (15%)	
White	1 (2%)	0	1 (5%)	p=NS
Location of furthest distant disease				
Inguinal LN	2 (5%)	0	2 (10%)	p = NS
High intraabdominal LN	2 (5%)	1 (5.5%)	1 (5%)	p = NS
Abdominal/liver/peritoneal	8 (21%)	2 (11%)	6 (30%)	p = NS
Lung	4 (10%)	3 (17%)	1 (5%)	p = NS
Mediastinal/Supraclavicular LN	17 (45%)	8 (44%)	9 (45%)	p = NS
Other (brain, bone)	5 (13%)	4 (22%)	1 (5%)	p = NS
Histology				
Squamous	32 (84%)	16 (89%)	16 (80%)	p = NS
Adenocarcinoma	6 (16%)	2 (11%)	4 (20%)	p = NS
Risk factors at diagnosis				
PCN at diagnosis^1^	13 (34%)	8 (44%)	5 (25%)	p=NS
On narcotics at diagnosis	22 (58%)	14 (77%)	8 (40%)	P=0.01
On anticoagulation^2^ at diagnosis	5 (13%)	4 (22%)	1 (5%)	p=0.11

ChemoRT, radio-sensitizing chemotherapy with radiation treatments; BMI, body mass index; LN, Lymph nodes; NS, nonsignificant; PCN, percutaneous nephrostomy tubes.

1. Due to limited access to urology and concern for stent failure due to tumor burden, PCN is the preferred method to control hydronephrosis related to cervical cancer in our institution. Only 1 patient in the cohort had a ureteral stent.

2. Therapeutic anticoagulation due to diagnosis with thromboembolism prior to treatments.

**Table 2:** Treatments received by stage IVB cervical cancer patients.

Treatment modalities	Total (N = 38)	No ChemoRT (N = 18)	ChemoRT (N = 20)	
Primary systemic chemotherapy				
Platinum with paclitaxel	38 (100%)	18 (100%)	20 (100%)	p = NS
Received bevacizumab	16 (42%)	7 (39%)	9 (45%)	p = NS
Received pembrolizumab	10 (26%)	7 (39%)	3 (15%)	p=0.09
Number of cycles (median, range)	6 (1-21)	6.6 (1-21)	5.75 (3-9)	p=NS
Time from diagnosis to first chemotherapy (days) (median, range)	57.5 (9-417)	65.5 (9-147)	45.5 (17-417)	p=NS
Other treatments				
Palliative Radiation^1^	8 (21%)	8 (55.6%)	0	p=0.008
Radiation to metastasis	5 (13%)	0	5 (25%)	p=0.02
Surgical Intervention^2^	8 (21%)	4 (22%)	4 (20%)	p=NS
Total Lines of Chemotherapy (median, range)	1 (1-4)	2 (1-3)	1 (1-4)	p = NS

ChemoRT, consolidative chemoradiation; NS, nonsignificant.

1. Indications for radiation: palliative for bleeding (6 patients), pain (2 patients), distant disease (hilar/mediastinal, brain, thoracic spine, liver, mediastinum, supraclavicular lymph node)

2. Indications for surgery: BSO for large ovarian metastases (4 patients), palliative GI procedure (PEG, loop ileostomy), palliative spine surgery, radical hysterectomy (2 patients).

**Table 3** presents characteristics of radiation and surgical treatments received by the patient cohort. Twenty patients received consolidative chemoradiation. The median time to complete radiation was 50.5 days (IQR 43-52.5). All but one chemoradiation patient received radiosensitizing chemotherapy, with a median number of cycles of 4 (IQR 3-4). The patient who did not receive chemotherapy was part of a clinical trial evaluating niraparib as a radiosensitizing agent. Seven patients (35%) were unable to tolerate 3 doses of radiosensitizing chemotherapy, most commonly due to thrombocytopenia. Sixteen patients (80%) underwent brachytherapy treatments, with the other 4 having a cervical radiation boost. At the time of radiation treatments, 5 patients (25%) had residual disease outside of the radiation field; these patients received stereotactic radiation to hilar/mediastinal lymph nodes, supraclavicular lymph nodes, or spinal metastases during or after consolidative pelvic radiation. Patients who received consolidative chemoradiation had a median overall survival of 26.7 months (IQR 16.8 – 55.9, 95%CI 9.9- 104.3) and time off treatment of 13.7 months (IQR 5.3-36.5, 95%CI 5.3 – 36.5). At last follow up, 7 patients (35%) had no evidence of disease (**Table 4**). Fifteen patients (75%) did receive subsequent chemotherapy, 11 patients for a disease recurrence that included disease withing the radiation field.

**Table 3:** Consolidative radiation treatment (N = 20).

ChemoRT patient attributes	
Residual disease at time of ChemoRT	
None	3 (15%)
Cervix only	5 (25%)
Pelvic/para-aortic LN	8 (40%)
Other	4 (20%)
Radio-sensitizing Chemotherapy	
Cisplatin	16 (80%)
Carboplatin	3 (15%)
Number of doses (median, IQR)	4 (3-4)
Fewer than 3 doses	7 (35%)
Due to thrombocytopenia	6
Due to patient preference	1
Radiation treatments	
Time for treatment (days) (median, IQR)	50.5 (43-52.5)
Brachytherapy	16 (80%)

ChemoRT, consolidative chemoradiation; LN, lymph nodes.

**Table 4:** Patient survival.

Patient survival	Total (N = 43)	No ChemoRT (N = 18)	ChemoRT (N = 25)	
Overall survival from diagnosis to last follow up (months) (median, IQR)	17.3 (10.7-32.6)	14 (9.1-17.6)	26.7 (16.8-55.9)	p=0.01
Treatment-free interval after initial chemotherapy or chemoradiation (months) (median, IQR)	6.5 (2.1-18.0)	1.9 (0.9-6.1)	13.7 (5.3-36.5)	p=0.01
Overall survival at 2 years	15 (38%)	2 (11%)	12 (60%)	p <0.01
Status at last follow up				
Died of disease/hospice	26 (68%)	17 (94.5%)	9 (45%)	p=0.0011
Alive with disease	4 (10%)	1 (5.5%)	3 (15%)	p=NS
No evidence of disease	7 (18%)	0	7 (35%)	p=0.0055
Died of other causes	1 (2.6%)	0	1 (5%)	p=NS

ChemoRT, consolidative chemoradiation; OS, Overall survival; NS, nonsignificant.

**Figure 1:**
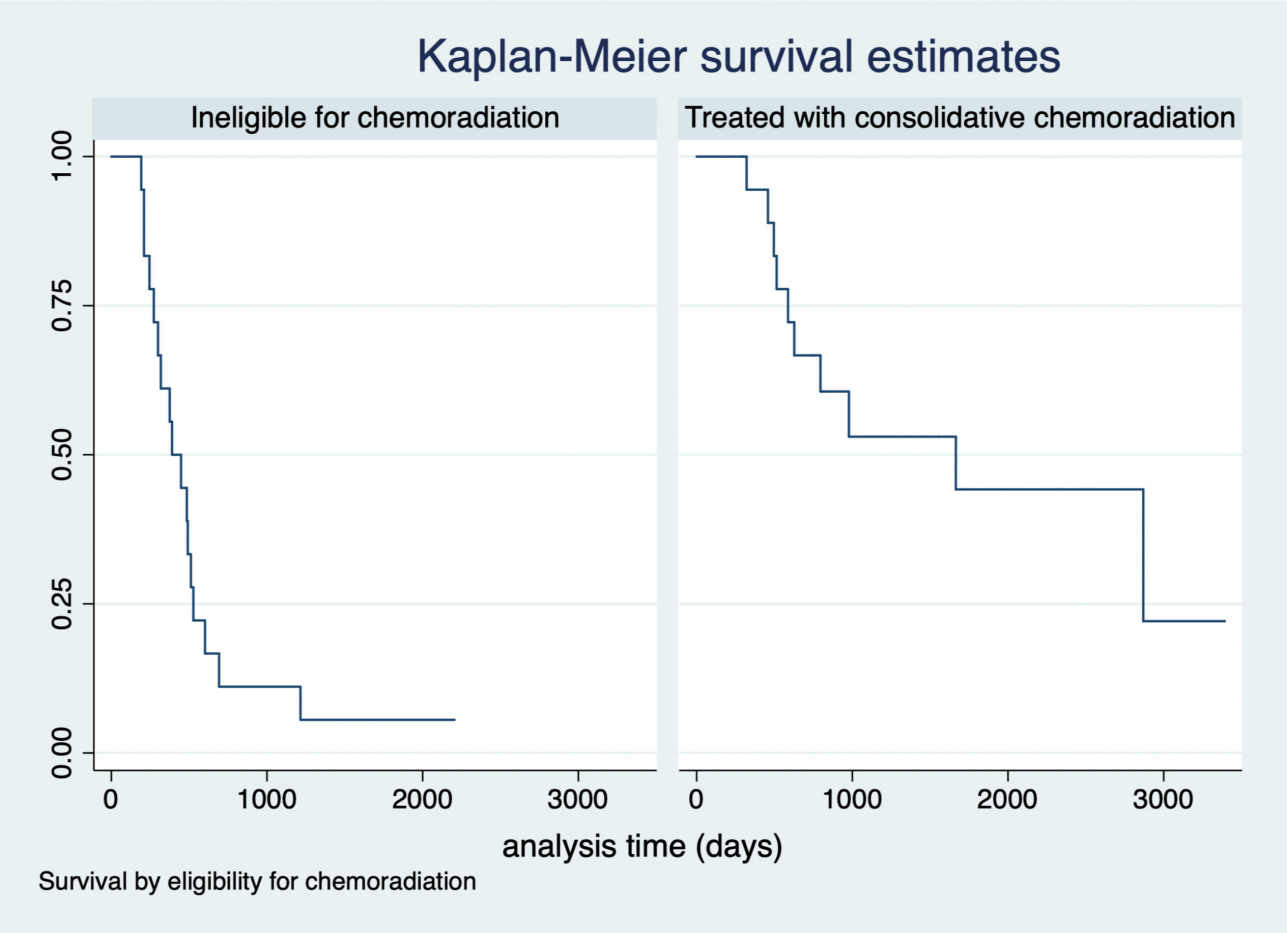
Survival analysis for patients ineligible for chemoRT and those who underwent consolidative chemoRT.

Eighteen patients (47%) had cancer progression prior to being eligible for chemoradiation and were treated with ongoing systemic treatments; these patients had a median overall survival of 14 months (IQR 9.1-17.6, 95%CI 6.4 – 73.5) (**Table 4**). Half of these patients succumbed to their disease prior to a second-line chemotherapy, and median time off treatment was 1.9 months (IQR 0.9-6.1, 95%CI 0.6-66.4). On evaluation with logistic regression, patients who progressed without becoming eligible for chemoradiation were more be on pain medications before treatment (OR 0.17, p=0.059, 95%CI 0.03 – 1.06) and be anticoagulated for venous thromboembolism at diagnosis (OR 0.03, p=0.046, 95%CI 0.001-0.9) and have disease in the mediastinum/lungs (OR 0.44, p=0.04, 95%CI 0.21-0.95). Twenty-one patients had nodal metastases and 17 patients had visceral metastases; 57% and 47%, respectively, went on to chemoRT (p=0.55), with no difference in overall survival. Over 200 charts were screened from a search through the medical records to determine patients eligible for the study, and 38 patients were included (see **Figure 1**). **Figure 1** shows the survival analysis for both groups of patients, those who received radiation and those who were not eligible for radiation.

## 4 Discussion

About half of our patient cohort had a sufficient response to treatment of distant disease to be eligible for consolidative chemoradiation, with a goal of improving pelvic control, cancer-related symptoms such as pain and need for ureteral diversion, and time off treatments. There was substantial time off treatments, with a tail lasting many years without treatment in the setting of a diagnosis of an aggressive metastatic cancer. The other half of patients continued on chemotherapy or changed to second line chemotherapy and inherently had poor outcomes. Studies comparing chemotherapy alone versus consolidative radiation in matched patients are needed in order to compare outcomes for these interventions, as we would expect that these two cohorts to have vastly different outcomes, as seen in this study. The difference in outcomes is expected to be from the different disease biology between the two groups, those who respond and can have consolidative chemoradiation, and those who do not respond well to chemotherapy; it is not attributed to the chemoradiation itself. This trial focused on describing the two paths for stage IVB cervical cancer patients in our institution.

Chemotherapy continues to be the backbone of systemic treatments for metastatic cervical cancer, and immunotherapy is also becoming standard. There have been some notable improvements in outcomes with the addition of pembrolizumab. Keynote-826 showed overall improvement in progression free survival and overall survival with the addition of pembrolizumab to chemotherapy, with median overall survival and progression free survival of 18.6 and 10.5 months for patients with PD-L1 positive tumors (8). Notably, however, Keynote-826 and other trials for metastatic or recurrence cervical cancer such as GOG 240 were designed to continue systemic treatments until disease progression or intolerance, and patients were on treatment for a median of 10 months before changing to another type of chemotherapy (6). These trials do not include the possibility of consolidative radiation for patients with good extra-pelvic response to chemotherapy. Our study suggests that in this select patient population, chemoradiation may allow time off treatment, which ongoing chemotherapy generally does not allow. Direct comparison studies are needed to know if chemoradiation will affect survival times, and evaluation of quality of life during this time would also be critical.

Few of our patients received pembrolizumab because it had not yet been adopted into standard of care at the time many of these patients were treated. We would anticipate that the addition of pembrolizumab would make more patients eligible for chemoradiation, and that continuing pembrolizumab during radiation and as maintenance as per the Keynote A18 protocol would continue to improve outcomes (6). Additionally, immunotherapy can be potentiated by combining with radiation, suggesting that multi-modality treatments may continue to improve outcomes. Our study was not powered to evaluate the difference in outcomes between those who did and did not receive immunotherapy, but no stark signals were noted in the 10 patients who did receive immunotherapy.

Another recent study supporting the use combination of systemic chemotherapy followed by radiation is the Interlace trial. This trial was for locally-advanced disease, as opposed to our stage IVB cohort, but it showed improved outcomes with induction chemotherapy followed by chemoradiation (6). While anticipated responses are lower for our more advanced patients, the new results of the Interlace trial support the effectiveness of combining induction chemotherapy and chemoradiation in cervical cancer.

Our retrospective study does show that about a third of patients on chemoradiation were not able to tolerate at least 3 cycles of concurrent platinum-based chemotherapy due to bone marrow toxicity or performance status. The Interlace trial with weekly induction chemotherapy also found 17% of patients unable to tolerate at least 4 cycles of sensitizing chemotherapy (6). It is not surprising that more advanced patients with longer induction treatments would have low bone marrow resilience. It is best to push for all concurrent chemotherapy cycles, however outcomes with the radiation were similar to the Keynote-826 trial; at last follow up in our cohort, 9 patients (21% of the total patients) had no evidence of disease and were off treatment, similar to the 22% complete response rate in Keynote-826 (8). However, as previously noted, patients on Keynote-826 did not get a break from treatments. Our patients had a median of almost 8 months treatment-free time, with a tail for those who didn’t recur of up to 49 months.

In addition to chemotherapy for advanced cervical cancer treatment, radiation is being used more often in the metastatic setting. Stereotactic body radiation therapy (SBRT) in particular is for oligometastatic disease in gynecologic cancers (14–16). Targeting residual disease after general response to systemic treatment for cervical cancer has not been evaluated in large studies but can provide substantial symptom control and even time off systemic treatments. The role of consolidative chemoradiation after response of distant metastases to induction chemotherapy can be viewed through a similar lens.

Additionally, cervical cancer is a cancer that causes many symptoms, particularly pelvic pain, ureteral obstruction requiring urinary stenting or diversion, fistulas and bowel obstructions. These heavily affect quality of life. In our institution, patients with a good response to 3–6 cycles of systemic therapy and resolution of extra pelvic disease are recommended to undergo consolidative chemoradiation to the pelvis to help alleviate cancer-related symptoms, improve local control, and provide a treatment break at the end of the radiation treatments. The patients who are eligible for chemoRT are by definition already good responders destined for improved outcomes. Chemoradiation provides an option to maximize response and allow time off chemotherapy. Anecdotal experience suggests that it can also help with quality of life in terms of cancer-related pain and need for urinary diversion, although this study did not assess these outcomes.

Lastly, predicting who can respond to chemotherapy and be eligible for chemoRT can be helpful in patient counseling and patient selection. As we can see in **Figure 1**, the Kaplan Meier curves for these cohorts was very different. We would expect a difference in outcomes even in the setting of ongoing chemotherapy, as those who respond well to first-line chemotherapy generally have favorable prognosis compared to those who progress through first-line chemotherapy. It would be helpful for patient counseling if we could predict the likely survival curve for an individual patient based on clinical criteria at the time of diagnosis. Unfortunately, in our patient cohort of stage IVB patients, only narcotics at the time of diagnosis was statistically significant; histology, visceral versus nodal metastases, age, race, language, and size of cervical mass were not statistically significant factors, and anticoagulation at time of diagnosis was only had borderline significance. More studies will be needed to elaborate predictive factors of response to chemotherapy in this patient population.

This study has limitations. First, it is a retrospective study with the limitations implicit in retrospective analyses, especially its limited ability to control for confounding variables. There is no comparison group of patients who responded well to chemotherapy but did not undergo consolidative radiation, and so it is difficult to assess whether they would have had similar time off chemotherapy or survival outcomes. Additionally, many of these patients started treatments before immunotherapy was used concurrently with chemotherapy for metastatic cervical cancer, and so outcomes of a more modern cohort may have different outcomes. There is also a lower than expected rate of use of bevacizumab in our patient cohort. GOG 204 showed that the addition of bevacizumab to chemotherapy for advanced or recurrent cervical cancer improved overall survival by 3.7 months (17). However, there is a practice bias against bevacizumab use in our safety net hospital due to experienced high rates of fistulas and other complications, which explains the lower rate of use for our patients. Additionally, as we consider consolidative chemoradiation for all metastatic patients if they respond to chemotherapy, avoiding bevacizumab can limit the expected complications of radiation after bevacizumab. The rate of bevacizumab use was the same between those who were eligible for chemoRT versus those who progressed, so we would not expect increased use overall to make a substantial difference. Lastly, the small sample size limits statistical power and generalizability of the results. This is a patient population at a safety-net hospital, frequently with difficulty with access to care and follow up. For example, there are patients in our data set who waited more than a year between their biopsy and their treatment due to financial or social barriers to treatments during that time, which we hope is not common in other patient cohorts.

## 5 Conclusions

We found that patients with stage IVB cervical cancer responsive to chemotherapy who subsequently underwent chemoradiation had lasting progression free survival and could have months off systemic treatments. Further research is needed to identify optimal chemoradiation regimens for local disease control and improved health outcomes in the management of metastatic cervical cancer, particularly given the increasing use of pembrolizumab as maintenance therapy.

## Data Availability

Datasets available upon request. The raw data supporting the conclusions of this article will be made available by the authors, without undue reservation.
